# Multimodal Distribution of Human Cold Pain Thresholds

**DOI:** 10.1371/journal.pone.0125822

**Published:** 2015-05-20

**Authors:** Jörn Lötsch, Violeta Dimova, Isabel Lieb, Michael Zimmermann, Bruno G. Oertel, Alfred Ultsch

**Affiliations:** 1 Institute of Clinical Pharmacology, Goethe—University, Theodor-Stern-Kai 7, 60590, Frankfurt am Main, Germany; 2 Fraunhofer Institute for Molecular Biology and Applied Ecology IME, Project Group Translational Medicine and Pharmacology TMP, Theodor-Stern-Kai 7, 60596, Frankfurt am Main, Germany; 3 Department of Anesthesiology, Intensive Care Medicine and Pain Therapy, University Hospital Frankfurt, Theodor-Stern-Kai 7, 60590, Frankfurt am Main, Germany; 4 DataBionics Research Group, University of Marburg, Hans-Meerwein-Straße, 35032, Marburg, Germany; University of Ottawa, CANADA

## Abstract

**Background:**

It is assumed that different pain phenotypes are based on varying molecular pathomechanisms. Distinct ion channels seem to be associated with the perception of cold pain, in particular TRPM8 and TRPA1 have been highlighted previously. The present study analyzed the distribution of cold pain thresholds with focus at describing the multimodality based on the hypothesis that it reflects a contribution of distinct ion channels.

**Methods:**

Cold pain thresholds (CPT) were available from 329 healthy volunteers (aged 18 – 37 years; 159 men) enrolled in previous studies. The distribution of the pooled and log-transformed threshold data was described using a kernel density estimation (Pareto Density Estimation (PDE)) and subsequently, the log data was modeled as a mixture of Gaussian distributions using the expectation maximization (EM) algorithm to optimize the fit.

**Results:**

CPTs were clearly multi-modally distributed. Fitting a Gaussian Mixture Model (GMM) to the log-transformed threshold data revealed that the best fit is obtained when applying a three-model distribution pattern. The modes of the identified three Gaussian distributions, retransformed from the log domain to the mean stimulation temperatures at which the subjects had indicated pain thresholds, were obtained at 23.7 °C, 13.2 °C and 1.5 °C for Gaussian #1, #2 and #3, respectively.

**Conclusions:**

The localization of the first and second Gaussians was interpreted as reflecting the contribution of two different cold sensors. From the calculated localization of the modes of the first two Gaussians, the hypothesis of an involvement of TRPM8, sensing temperatures from 25 – 24 °C, and TRPA1, sensing cold from 17 °C can be derived. In that case, subjects belonging to either Gaussian would possess a dominance of the one or the other receptor at the skin area where the cold stimuli had been applied. The findings therefore support a suitability of complex analytical approaches to detect mechanistically determined patterns from pain phenotype data.

## Introduction

Pain is a heterogeneous multifactorial sensation evolving from many molecular pathways [1] forming a complex pathophysiology [2]. On this complex molecular background, an increasing number of distinct pain phenotypes is being recognized. For example, neuropathic pain becomes regarded as a heterogeneous syndrome varying among subjects in positive and negative symptoms, which probably reflects different pathomechanisms [3]. Careful assessments of pain phenotypes therefore may serve as a window on underlying pathomechanisms [4]. This opens up new opportunities for the development of specific pain treatments as recently shown for painful diabetic neuropathy [5].

Thus, strategies to assess pain need to be re-evaluated with respect to their ability to reflect involved pathomechanisms and their molecular backgrounds. For example, pain thresholds to cold thermal stimuli, which are an integral component of modern pain test batteries [6,7], are regarded as single homogeneous pain measures. This contrasts to the complex mechanisms of low temperature perception mediated by several different ion channels [8,9], including TRP channels (e.g. TRPA1, TRPM3, TRPM8, TRPV1), Ca^2+^-activated Cl^-^ channels (e.g. ANO1), Ca^2+^-permeable ORAI1 ion channels, two-pore-domain K^+^ channels (e.g. KCNK2, KCNK4 and KCNK10) and voltage-gated Na^+^ channels (e.g. SCN10A). All of them have response maxima at different temperatures.

While molecular mechanisms support multi-modality of cold pain thresholds, cold pain thresholds are usually treated statistically as if originating from a simple unimodal distribution. However, this is sharply contrasted by the obvious multimodality of the distribution of cold pain thresholds (for example, see Fig 2 in [3]). The present analysis therefore addressed the distribution of cold pain thresholds with a focus at describing its multimodality. This was based on the hypothesis that a contribution of several distinct ion channels might be reflected in the distribution of data from cold pain thresholds.

## Methods

### Data origin and assessments of cold pain thresholds

The assessments followed the Declaration of Helsinki and were approved by the Ethics Committee of the Goethe University, Frankfurt am Main, Germany. Only healthy volunteers were included and informed written consent was obtained from each participating subject. The subjects’ state of health was assessed by medical history and physical examination, including vital signs. Exclusion criteria were a current clinical condition affecting pain, any other actual diseases, including current psychological or psychiatric disorders and intake of drugs, except for oral contraceptives, during the previous week.

Cold pain threshold data were obtained from a total of 329 healthy volunteers (aged 24.8 ± 3.1 years, mean ± standard deviation, range 18–37 years; 159 men). Data had been acquired for several pain measures to mechanical, thermal, electrical or chemical stimuli; additional olfactory or psychological tests were applied in two or one study, respectively. An overview about the studies from which the data originates in given in Table 1. Subjects who had participated in more than one study were only included once and were assigned to the first study in which they participated. All measurements were made in a single laboratory, using the same equipment under identical experimental conditions by five different postgraduate students (two in study [10,11] and each one in the other three studies [12–14]) under the supervision of the same senior researchers (JL, BGO). The cold pain thresholds on which the focus of the present analysis was laid had been reported previously together with results of other tests in a non-redundant manner [11–14].

**Table 1:** Brief overview of the studies, from which cold pain threshold data were used for the present analysis.

Study	No. subjects enrolled	No. subjects presently analyzed *	Pain assessments	Other psycho(physical) assessments	No. investigators	Data subset
[11]	122	122	Pain thresholds to mechanical (punctate and blunt pressure), thermal (heat and cold) electrical (5 Hz 0–20 mA sine waves) and chemical (intranasal CO_2_) stimuli.		2^‡^	#1
						#2
[12]	75	70		Olfactory tests (odor thresholds, odor discrimination, odor identification).	1	#3
[13]	84	83			1	#4
[14]	110	54	Pain thresholds to mechanical (punctate and blunt pressure), thermal (heat and cold) electrical (5 Hz 0–20 mA sine waves) and chemical (intranasal CO_2_) stimuli; Additional application of a standardized quantitative sensory test battery QST [7].	Test of psychological parameters related to mood, somatization and state anxiety, dispositional optimisms, catastrophizing, pain anxiety and vigilance.	1	#5

*: The number of 329 non-redundant subjects presently analyzed is smaller than the sum of the subjects enrolled in the four studies from which the data originate, as while some subjects had participated in more than one study each subject was included only once.

^‡^: Because of two main observes, the present analysis plan had predefined a split of the study data into two subsets to exclude potential inter-observer differences [15–17], which would not have applied to the other three studies.

Pain thresholds to cold stimuli were assessed using a Thermal Sensory Analyzer (Medoc Advanced Medical Systems Ltd., Ramat Yishai, Israel). Stimuli were applied with a 3 x 3 cm^2^ thermode placed in a randomized manner on the skin of the left or right volar forearm. The temperature was lowered from a baseline temperature of 32°C to a cut-off temperature of 0°C at a constant speed of 1°C/s. The subjects were instructed to press a button at the first sensation of pain. Threshold measurements were repeated five times. According to previous experiments during method setup, the median value, as a more robust measure than the arithmetic average, of the single measurements [13,18] was taken as the cold pain threshold (CPT).

### Data analysis

Statistics were performed using SPSS (version 22 for Linux, IBM SPSS Inc., Chicago, USA) and Matlab (version 8.3.0.532 for Linux MathWorks, Natick, MS, USA); the α level was set at 5%. Data was analyzed separately according to the cases measured by single researchers. This resulted in five subsets, because in one study [11] two experimenters had been involved. This one data set per observer treatment accommodated for potential inter-observer effects, which are occasionally described in the context of pain influences of the subject’s or the observer’s gender [15–17]. Analyses were performed by an information scientist (AU) who at the time was unaware of the underlying molecular hypothesis. In a first step, the comparability of data subsets was explored by means of analysis of variance (ANOVA) with between-subjects factors “sex” (one degree of freedom, df) and “subset” (df = 4), and “age” as covariate. In addition, the modality of the distribution of the cold pain thresholds was visually explored, separately for data subsets and the subjects’ sexes. The relationship between increasing perception and increasing stimulus strength was evaluated by redefining the baseline temperature of 32°C as zero stimulus intensity and the highest possible intensity as 0°C. This provided an axis of increasing stimulus intensity (SI), calculated as *SI* = *32°C—CPT*, on which the distribution of the cold pain thresholds, CPT, was analyzed. Truncated data acquired occasionally due to the cut-off of the stimulation temperature at 0°C were extrapolated. Specifically, Data on cold pain thresholds were truncated at a cold pain threshold, *CPT* ≥ 0°C due to the technical cut-off of the cold stimulation device. Therefore, the lowest stimulus intensity was obtained at 32°C (SI = 32°C - 32°C = 0) and the highest at 0°C (SI = 32°C - 0°C = 32). N = 76 observations of this kind were made. To consistently extrapolate beyond this limit, a Gaussian Mixture Model (GMM) was fitted to the stimulus intensity data, given as *SI = 32°C - CPT*, using four Gaussians and optimizing the model using the EM algorithm. Using this GMM, extrapolated data points beyond the limit were randomly chosen from randomly generated data. The resulting data were tested for homogeneity with the theoretical distribution of GMM using a χ^2^ test (S1 Fig).

To accommodate the law of Weber and Fechner [19], *SIs* were zero invariant log-transformed to *LogSI = ln(SI+1)* [20]. This was followed by the estimation of the probability density function (PDF) of *LogSI* values using the Pareto Density Estimation (PDE). PDF represents the relative likelihood of a given continuous random variable taking on specific values. The PDE is a kernel density estimator particularly suitable for the discovery of mixtures of Gaussians [21]. The PDE analysis indicated a multimodal distribution for both SI and *LogSI*. The log-transformed data was subsequently modeled as a mixture of Gaussian distributions. Specifically, a Gaussian mixture model (GMM) is a weighted sum of *M* component Gaussian densities as given by the equation

$$p(x)=\;\sum_{i=0}^{M}w_{i}N(x\text{|}m_{i},s_{i})=\sum_{i=1}^{M}w_{i}\cdot \frac{1}{\sqrt{2\pi s_{i}}}\cdot e^{-\frac{{\left(x-m_{i}\right)}^{2}}{2s_{i}^{2}}}$$ 1

where *N(x|m*_*i*_, *s*_*i*_ *)* denotes Gaussian probability densities (components) with means *m*_*i*_ and standard deviations, *s*_*i*_. The *w*_*i*_ are the mixture weights indicating the relative contribution of each component Gaussian to the overall distribution, which add up to a value of 1. *M* denotes the number of components in the mixture. The parameters of the GMM were optimized using the expectation maximization (EM) algorithm [22]. To determine the optimum number of components, model optimization was done for *M* = 1 to 9 components. The quality of the obtained models was compared among different numbers of mixes using the averaged test statistic for χ^2^ goodness-of-fit test and a Scree test [23]. Subsequently to the identification of the value of *M*, the Bayes’ theorem was used to assign the *LogSI* values to *M* classes, *c*_*i*_, *I = 1*,*…*,*M*, of cold pain thresholds. Means, *m*_*i*_, and Bayes decision limits (Si values separating the Gaussians), of the optimum GMM were retransformed to original *SI* scale in t°C for further interpretations in terms of *CPT*. Finally, sex differences were tested with respect to classes of pain thresholds using χ^2^ statistics.

## Results

**Fig 1:**
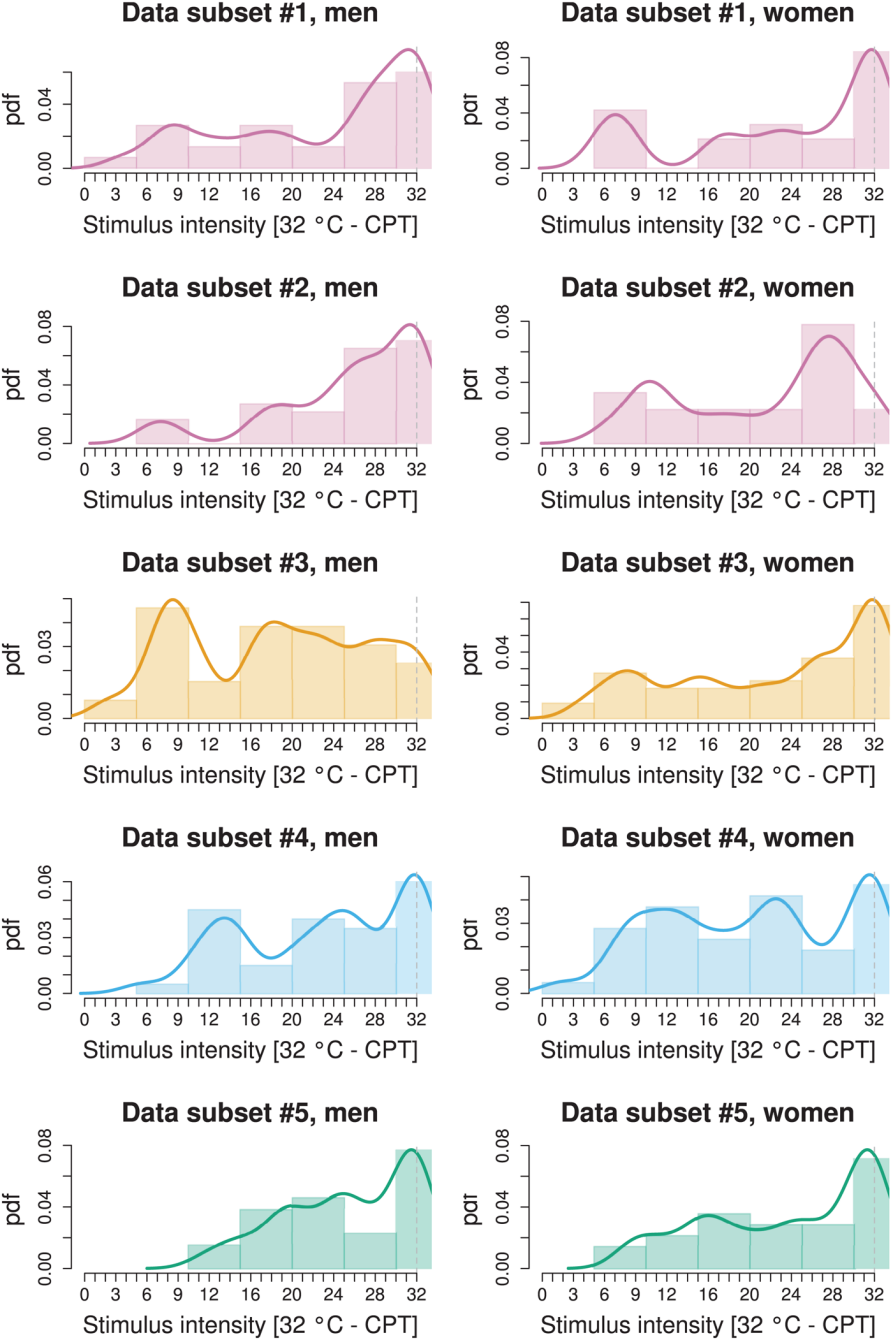
Distribution of cold pain thresholds as observed in the five different data sets (rows) corresponding to the four studies [11–14] as in study #1 [11], data was separated to accommodate the involvement of two observers in contrast to the other three studies where only a single observed had acquired the data (Table 1). The four different studies are drawn in different color to enhance the association of data subsets with the study in which they have been acquired. The graph displays the data after rescaling for stimulus intensity as *SI = 32°C - CPT* to provide increasing stimulus intensity along the abscissa with increasing x. The lower limit of the applied stimulus intensity by the Thermal Sensory Analyzer is marked with a perpendicular dashed line. Data is shown as histograms and superimposed probability density functions (pdf, Gaussian kernel), separately for men and women (columns). For the main analysis, all data subsets shown here were pooled, log-transformed and mathematically modeled for multi-modality (Fig 3).

**Fig 2:**
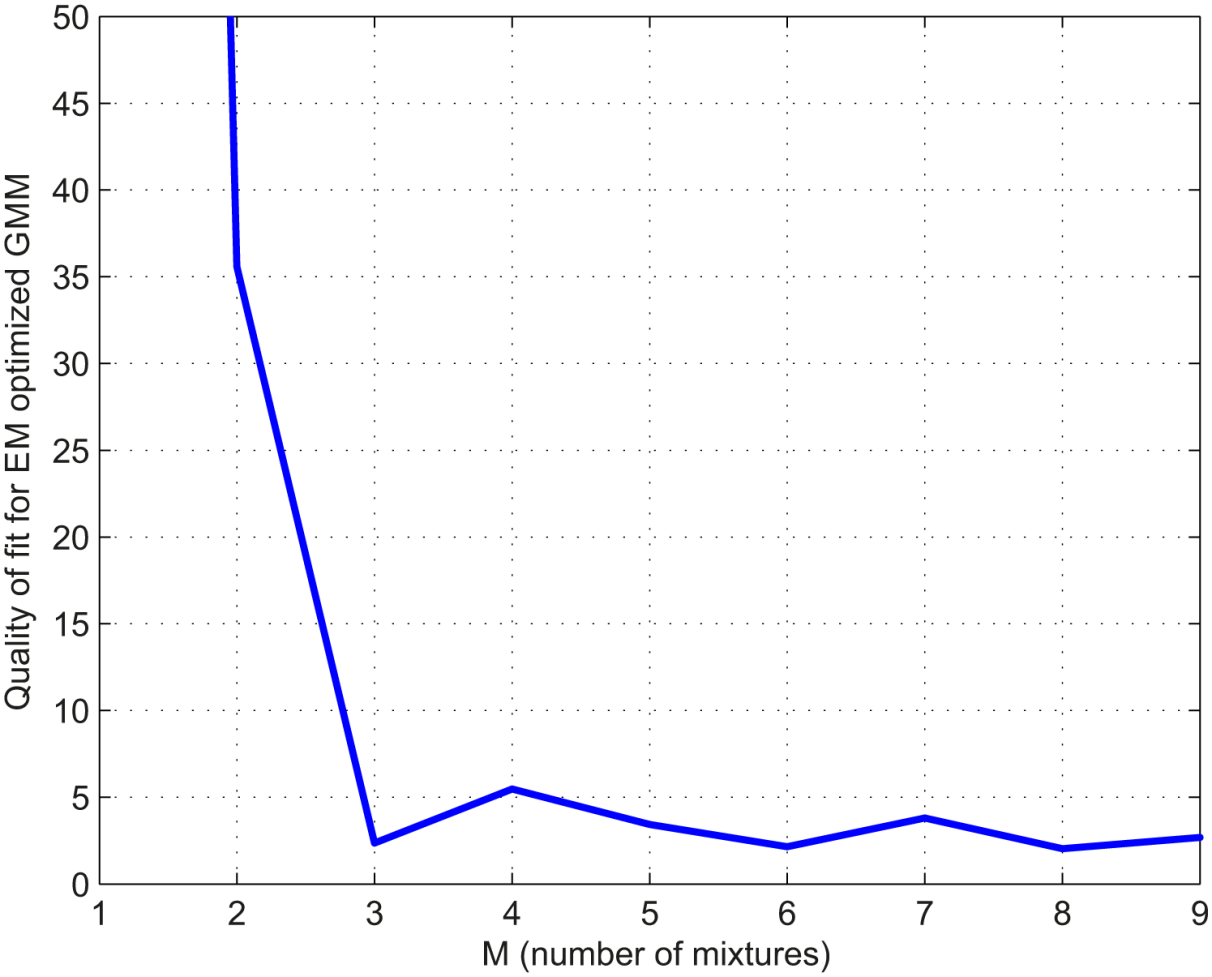
Scree plot [23] of the model quality for the EM fit of the Gaussian mixture, illustrating the number of components which should be assessed in order to explain a high degree of variation in the data. The plot clearly indicated that less than a mixture of three Gaussians provided an inadequate fit and more than three Gaussians did not further improve the fit.

Cold pain threshold (CPT) data comprised of n = 49, 73, 70, 83 and 54 non-redundant subjects according to the five data subsets, respectively. The data subsets had similar sex distributions among subjects (χ^2^ test: p > 0.1). Cold pain thresholds did not differ with respect to the subjects’ sex (ANOVA: F = 0.834, p > 0.05), age (F = 0.656, p > 0.05) and the assignment to the study subset (F = 2.343, p > 0.05). Visual inspection identified non-Gaussian distributions of CPTs in all separate data subsets (Fig 1). The distribution of the pooled n = 329 *LogSI* values, representing log transformed cold pain thresholds, could be described with a Gaussian mixture model composed of *M* = 3 Gaussians. This number of components for the mixture was found to be the best (χ^2^ goodness-of-fit test: p = 0.995 for non-correspondence of the model with the observed data; numerical parameter results in Table 2), with respect to model quality and simplicity (Fig 2).

**Table 2:** Values of variables obtained following modeling of the cold pain thresholds (rescaled for stimulus intensity, *SI = 32°C - CPT* to accommodate the increasing perception with increasing stimulus strength and zero-invariant log-transformed as *LogSI = Ln(SI+1)*), by means of the Gaussian mixture model (GMM given as $p\left(x\right)\;=\;\sum_{i\;=\;0}^{M}w_{i}N(x|m_{i},s_{i})$, for which the optimum number of mixes was found to be *M* = 3 (Figs 2 and 3), where *m*_*i*_, *s*_*i*_ and *w*_*i*_ are the parameters mean, standard deviation and relative weight of each of the Gaussians, respectively, obtained for the *LogSI* data.

	i = 1 (first Gaussian)	i = 2 (2^nd^ Gaussian)	i = 3 (3^rd^ Gaussian)
***m***_***i***_	2.235	2.9828	3.4495
***s***_***i***_	0.2246	0.3241	0.2317
***w***_***i***_	0.1491	0.3296	0.5213

Due to the data transformations, retransformation of the modes to *CPT* values is thus obtained as *CPT = 32°C—e* ^*LogSI*^ *+ 1*. This retransformation of the *m*_*i*_ values provides the modes of the three Gaussians in the linear temperature range over which *CPT* was measured, i.e., 23.6, 13.3 and 1.5°C for Gaussian number i = 1, 2 and 3, respectively.

**Fig 3:**
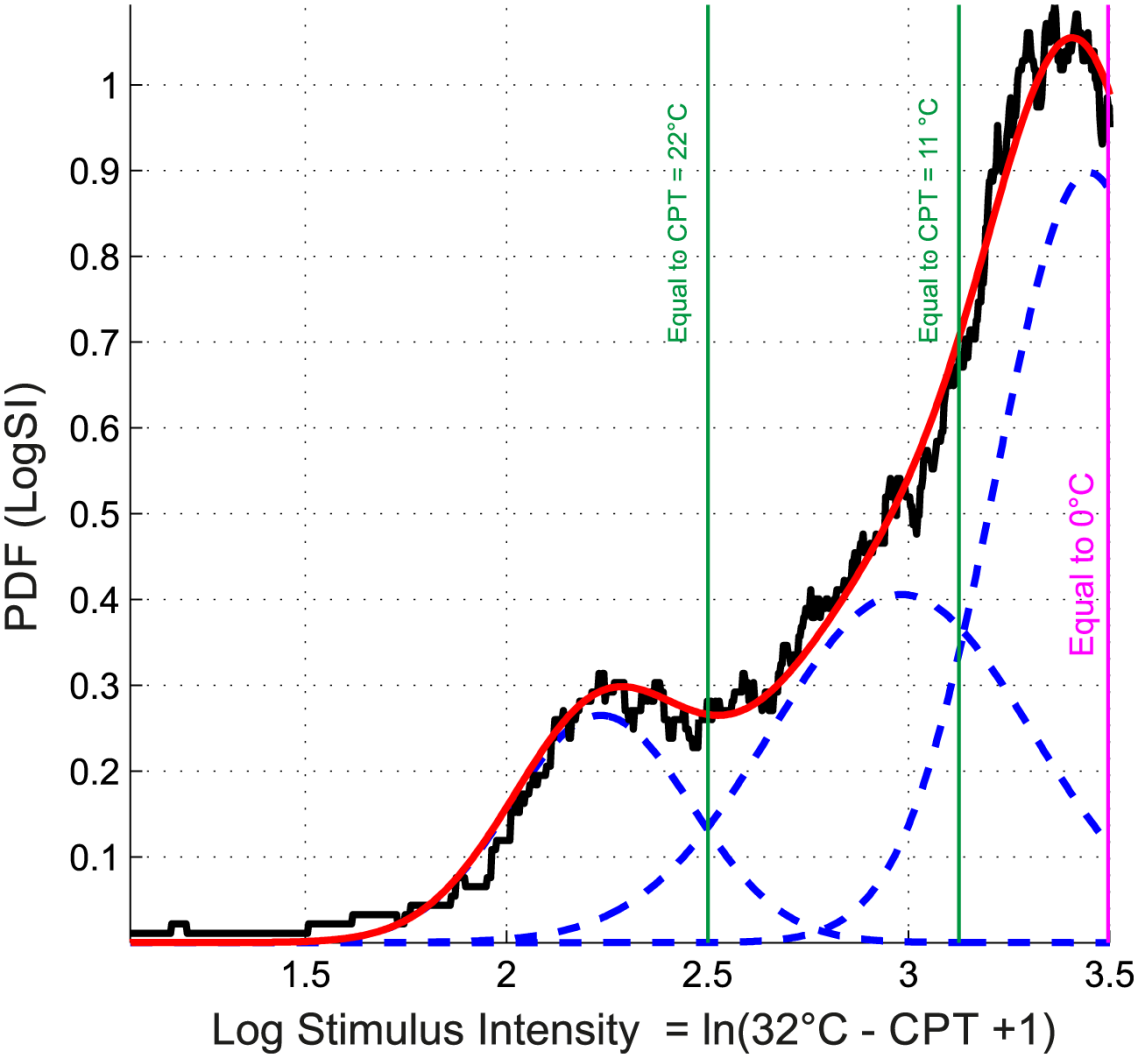
Distribution of the cold pain thresholds (*CPT*) observed in n = 329 subjects pooled from previous studies (Table 1). The graph displays the data after rescaling for stimulus intensity as *SI = 32°C - CPT* (Fig 1) and subsequent log transformation as *LogSI = ln(SI+1)*. The density distribution is presented as probability density function (PDF), estimated by means of the Pareto Density Estimation (PDE [21]). A Gaussian mixture model (Eq 1; GMM given as $p\left(x\right)\;=\;\sum_{i\;=\;0}^{M}w_{i}N(x|m_{i},s_{i})$), was fit to the data, for which the optimum number of mixes was found to be *M* = 3. Subject distribution among the obtained three Gaussians was n = 155, n = 61 and n = 113 for Gaussian 1–3, respectively, starting from the left.

The modes of these distributions, retransformed from the log domain to the mean stimulation temperatures at which the subjects had indicated pain thresholds, were obtained at 23.7°C, 13.2°C and 1.5°C for Gaussian 1, 2 and 3, respectively (Fig 3). The Bayesian decision limits indicating the temperatures separating the three Gaussians were found at 22°C, between the first and second Gaussian, and at 11°C between the second and the third Gaussian (Fig 3). The obtained subgroups of subjects displaying different cold pain sensitivities, i.e., belonging to either Gaussian 1, 2 or 3, did not significantly differ with respect to age (Kruskal-Wallis rank sum test: p > 0.1). With respect to sex, the observation that the first Gaussian, comprising subjects with the highest cold pain sensitivity, contained 8% more women than expected from their proportion in the whole pooled cohort, could not be statistically supported (χ^2^ test: p > 0.1). However, there was a significant difference with respect to the association of data subsets to the different Gaussian modes (χ^2^ test: p = 0.043). This was probably due to the comparatively higher proportion in subset #3 of subjects belonging to Gaussian 1 (30% versus 4–16% in the other subsets). This particular distribution was also suggested by a visual check of the original non-pooled data where a particularly high probability density was observed in the left part of the distribution of the 32°C - CPT data in the men of data set #3 (Fig 1). Indeed, the χ^2^ test became non-significant (p > 0.1) when excluding data subset #3. However, this particularity of data set #3 was not the cause of the present findings. When excluding this data set and reanalyzing the pooled data by means of GMM, the tri-modality prevailed (see S2 Fig).

## Discussion

A multimodal distribution of cold pain threshold (CPT) data was observed in all data subsets and also resulted from the analysis of the pooled data. The observed multimodality seems to be highly characteristic, as it is also clearly evident in independent data sets, for instance, it is visible in the cold pain thresholds data from 1236 neuropathic pain patients and 180 controls (see Fig 2, upper right panel, page 443 in reference [3]). Therefore, current data processing strategies, e.g., [6], which treat cold pain thresholds as if they would originate from a simple molecular background should be revised to reflect the multimodal distribution.

Cold pain thresholds at 24°C or higher have not always been obtained when assessing human cold pain sensitivity. For example, in a small cohort of six or five subjects assessed in two experiments, stimuli at 22 or 16°C were not perceived as painful [24]. However, the present observation of CPTs at 24°C (the mode of the first Gaussian) and above agrees well with other observations obtained in much larger data sets. For example, in 180 healthy young men or women [25] the upper 95% confidence interval limits of CPTs were identified at temperatures of 27.21 or 29.62°C, respectively (see Table S1 of [25]; values obtained at the hand and in subjects aged 20–30 years), which implies the observation of such temperatures in some subjects. However, this particular subgroup of subjects seems to be small. In the present data, thresholds of ≥24°C were seen in 24 subjects (7.3%). In the first Gaussian with a mode at 24°C were 12.8% of the subjects, hence, half of them (6.4%) must have had a threshold ≥ 24°C, which well agrees with the raw observation. The same fraction also results from re-analyzing the reported data of [25]. That is, with arithmetic means and standard deviations of 11.24 ± 8.15 in men and 15.61 ± 7.15°C in women (see Table S1 of [25]), which assumes a normal distribution, the probability of subjects with thresholds ≥24°C can be obtained as the area under the Gauss curves beyond this temperature. This calculation resulted in a probability of 5.9 and 12% for men or women, respectively, to display thresholds ≥ 24°C. This well agrees with the present data although the calculation has to be regarded with reservations as it shows a multi-modality of CPTs, which is incompatible with a report of means and standard deviations from the whole cohort and the derived calculations. This repeatedly observed small fraction of subjects with these comparatively low CPTs (probability of about one in 10–20 subjects) is probably also the reason for their non-observation in a cohort of 11 subjects [24]. However, a particular sensitivity to cold, possibly met in these subjects, might be of clinical importance. Cold hyperalgesia or allodynia have been highlighted as a striking symptom in patients with neuropathic pain [26,27]. Of 465 patients with different neuropathies, 9% were characterized by cold pain hyperalgesia [28] indicating a sensitization of cold nociceptors [29]. In a small study with 18 nerve injured patients, reduction of paroxysmal pain, tactile and cold allodynia was reported following treatment with gabapentin [30]. As the multimodality of cold pain thresholds had not been explored, it cannot be deduced from published data whether this symptoms are particularly prominent in patients with a low CPT or whether the probability of CPTs at ≥ 24°C increased in these patients. Hence, the importance of future assessments of possible similarities between the here reported modes of CPT with subgroups of neuropathic pain patients.

The relatively rare occurrence of subjects with CPTs already at higher temperatures may also occasionally trigger the recruitment of relatively more such subjects in a study. This might have been the case with the present data subset #3. Specifically, although all data subsets were comparable with respect to the subject’s age, sex and the observed thresholds to cold pain as established by the non-significance of the respective covariates of factors in the ANOVA, the association of subjects to the different Gaussian modes differed significantly for the different data subsets. This could be tracked to data subset #3, where probably a higher fraction of subjects with CPTs around 24°C had been recruited. Nevertheless, the obtained results still reflect a general characteristic of cold pain thresholds, which is supported by the non-significance of the data subset difference and the prevalence of the tri-modality (see S2 Fig) when leaving out subset #3.

Based on the above-mentioned observation of a distinction of cool and noxious cold perceptions in 11 subjects, the hypothesis has been raised that these sensations are mediated via different afferent channels [24]. However, the present data, as well as independent observations [25], show that temperatures of 16 or 22°C as used in [24] can evoke pain and not only cool sensations. Therefore, the conclusion is suggested that psychophysical responses to cold stimuli reflect an even more complex pathophysiology. As the subjects’ sex or study origin provided no simple interpretation of the multi-modality of the CPT distribution, the hypothesis of an involvement of several distinct thermo-sensors in the perception of cold pain arises. In particular, the modes of the first two Gaussians are highly suggestive of the activation of two well-known thermosensors. Specifically, the temperature range of 25–24°C over which TRPM8 ion channels start to sense cold [31] is likely to have caused the first Gaussian with a mode at 24°C. Similarly, the temperature of 17°C at which TRPA1 ion channels start to sense cold [32] fits well with the occurrence of the second Gaussian with a mode at 15°C. Based on this hypothesis of an involvement of TRPM8 or TRPA1, the classification of subjects into either the first or the second Gaussian might reflect the relative importance of TRPM8 versus TRPA1 in their individual sensitivity to cold pain. That is, subjects in the first Gaussian might have a dominance of TRPM8 at the skin area where the stimuli have been applied, whereas in subjects assigned to the second Gaussian the dominance is shifted to TRPA1. Such allocation would accommodate the reported complexity of cold sensation at the neuronal level [33].

In contrast to the first two Gaussians, which with n = 216 subjects comprised two thirds of the cohort, the interpretation of the third distribution is less evident. Below temperatures of 10°C, specific cold pain sensing channels have not yet been defined. Further known cold sensors qualify as candidates, such as TRPC5 which, however, is sensitive at temperatures of 37–25°C [34], or others that have been hypothesized, such as potassium channels (KCNK2) implicated in neuropathic pain [35], Na^+^/K^+^ adenosine triphosphatases [36] proposed with reference to pain [37] or acid sensing ion channels (ASIC2 and ASIC3 [38]). Regardless of the origin of the third Gaussian and even when truncated data had been excluded, the main interpretation is still supported by the first two Gaussians.

The present proposal to group subjects for cold pain sensitivity according to a, still hypothetical, ion channel dominance has implications for analgesic drug development and personalized pain therapy. That is, subjects in either the first or second Gaussian would differently benefit from analgesic therapies using either TRPM8 or TRPA1 antagonists. This concept could also be exploited in drug development strategies for antagonists of these channels, which are among several therapeutic targets of interest http://www.nature.com/nrd/journal/v10/n8/fig_tab/nrd3529_T1.html. Specifically, the obtained grouping of subjects suggests the possibility to selectively enroll subjects with particularly high cold pain sensitivities mediate via either TRPM8 or TRPA1 as highly selected study cohorts during human phases of drug development, which can be expected to improve the predictivity of human experimental pain studies for clinical drug efficacy [39]. Moreover, from the present results also follows the possible utility of developing multimodal analgesics that antagonize both TRPM8 and TRPA1, which would cover both groups of subjects, provided that this dual mechanism of action is not associated with more side effects.

Modern complex pain test batteries explicitly [7] address characteristic constellations of sensory signs and symptoms under the assumption that they reflect the different pathophysiological mechanisms underlying pain generation [40]. The present analysis of cold pain thresholds suggests that molecularly plausibly hypothesis about the underlying mechanisms can be derived when the structural patterns and distributions of the data are taken into account. This approach is not restricted to cold pain. Many other nociceptive stimuli evoke pain via more than a single sensor; for example heat pain qualifies for similar approaches as the sensation of high temperatures is also mediated via several different thermosensors [8]. Thus, the findings presented here illustrate the likely suitability of advanced data analysis, with special regard to data distribution, for identifying potentially meaningful phenotypes. Importantly, while the present analysis clearly shows a multi-modality of the CPT distribution, the allocation of two particular ion channels is hypothetical and results merely from the interpretation of the presently observed localization of the Gaussian modes. A detailed analysis of the molecular background of multimodal distribution of CPTs requires the careful regard of candidate cold sensing ion channels.

## Supporting Information

S1 Dataset This file contains the source data as an ASCII text file providing the data as a matrix of subjects (rows) and variables (columns) with self-explain column headers. (TXT)

S1 Fig QQ-plot of the Cold data, calculated as 32°C—cold pain threshold (CPT) versus the Gaussian Mixture Model (GMM) together with the cut-off temperature. (EPS)

S2 Fig Plot of the distribution of the cold pain thresholds (CPT) analogously to Fig 3 in the main paper, however, excluding data subset #3. The graph displays the data after rescaling for stimulus intensity as *SI = 32°C - CPT* and subsequent log transformation as *LogSI = ln(SI+1)*. The density distribution is presented as probability density function (PDF), estimated by means of the Pareto Density Estimation (PDE).
(EPS)
